# Lymphocyte-to-monocyte ratio as a prognostic and potential tumor microenvironment indicator in advanced soft tissue sarcoma treated with first-line doxorubicin therapy

**DOI:** 10.1038/s41598-023-37616-w

**Published:** 2023-07-03

**Authors:** Sho Watanabe, Tatsunori Shimoi, Tadaaki Nishikawa, Asuka Kawachi, Hitomi Sumiyoshi Okuma, Momoko Tokura, Shu Yazaki, Chiharu Mizoguchi, Motoko Arakaki, Ayumi Saito, Shosuke Kita, Kasumi Yamamoto, Yuki Kojima, Kazuki Sudo, Emi Noguchi, Akihiko Yoshida, Akira Kawai, Yasuhiro Fujiwara, Kan Yonemori

**Affiliations:** 1 Department of Medical Oncology, National Cancer Center Hospital, 1-1, Tsukiji 5, Chuo-ku, Tokyo, 104-0045 Japan; 2 Division of Cancer Immunology, Exploratory Oncology Research and Clinical Trial Center, National Cancer Center East, 5-1, Kashiwanoha 6, Kashiwa, Chiba 277-8577 Japan; 3 Department of Pathology and Clinical Laboratories, National Cancer Center Hospital, 1-1, Tsukiji 5, Chuo-ku, Tokyo, 104-0045 Japan; 4 Department of Musculoskeletal Oncology, National Cancer Center Hospital, 1-1, Tsukiji 5, Chuo-ku, Tokyo, 104-0045 Japan

**Keywords:** Cancer microenvironment, Sarcoma, Translational research

## Abstract

Prognostic value of hematologic indices and their association with the tumor microenvironment (TME) remain unclear in advanced soft tissue sarcoma (STS). We aimed to evaluate their prognostic value and correlation with the TME status in advanced STS treated with first-line doxorubicin (DXR) therapy. Clinical data and three hematological indices, including lymphocyte-to-monocyte ratio (LMR), platelet-to-lymphocyte ratio, and neutrophil-to-lymphocyte ratio, were collected from 149 patients with advanced STS. The TME status was pathologically examined by CD3, CD68, and CD20 staining of resected tumor slides. In a multivariate Cox analysis, low LMR and absence of primary tumor resection were independently associated with worse overall survival (OS) (HR 3.93, *p* = 0.001; HR 1.71, *p* = 0.03). A prognostic model using these variables predicted OS with greater area under curves than those obtained using Systemic Inflammatory Score and Glasgow Prognostic Score. The LMR significantly correlated with the tumoral CD3/CD68-positive cell ratio in surgical specimens (R = 0.959, *p* = 0.04). In conclusion, LMR was a prognostic factor in advanced STS treated with first-line DXR therapy. LMR could partially reflect anti-tumor immunity in the TME and have the prognostic value. The potential role of LMR as an indicator of TME status warrants further investigation.

## Introduction

Soft tissue sarcoma (STS) is a rare, heterogeneous tumor of mesenchymal origin with an incidence of approximately 1% in adults. There are at least 100 different histological and molecular subtypes of STS that show variable clinical behaviors^1^. Except for subtypes with unique histological presentations, such as Ewing sarcoma or rhabdomyosarcoma that often affect children, treatment has not been individualized for most subtypes of STS. Despite advances in multidisciplinary treatment, the 5-year survival rate of patients with STS remains approximately 60%^2,3^. Furthermore, 25% patients develop distant metastasis after curative resection of the primary tumor^1^. Unfortunately, the prognosis of patients with metastasis is poor, with a 5-year survival rate of only 15%^3^. The standard first-line systemic therapy for advanced STS includes doxorubicin (DXR) monotherapy^4,5^. Phase 3 trials have shown a median progression-free survival (PFS) of 4–7 months and a response rate of 15–18% for patients treated with first-line DXR monotherapy^6,7^.

Prognostication of advanced STS is challenging. Patients treated with DXR therapy possess significantly different overall survivals (OS) with an interquartile range of 19.7 months (9.9–35.5 months), potentially due to the heterogeneity of STS^7^. Active agents beyond DXR with the capacity to treat certain histological subtypes are being developed^8–11^. Specifying accurate prognostic markers can help to predict patient outcomes, and, thus, facilitates individual treatment options. However, the current TNM staging system classifies STS according to tumor size, depth, nodal involvement, distant metastases, and malignancy grade^12^, and does not consider the histological diversity of STS^13,14^. Furthermore, this staging system is based on the outcomes of patients post-surgery and does not necessarily predict the survival of those treated with chemotherapy instead. An improvement in prognostic classification is warranted for monitoring patients with STS^15^.

As cancer alters immune system function^16^, immune cell counts can be used to predict patient outcomes. Certain hematological indices, such as the lymphocyte-to-monocyte ratio (LMR), neutrophil-to-lymphocyte ratio (NLR), and platelet-to-lymphocyte ratio (PLR), are derived from differential white blood cell (WBC) counts and reflect systemic immunity. The prognostic role of these indices has been examined in patients with STS pre-surgery as well as during a variety of treatments^17–31^; however, these studies have rarely examined the indices of patients prior to chemotherapy. Therefore, the performance of current prognostic models based on these studies may not be representative of patient outcomes after chemotherapy. Despite the prevalence of advanced STS^1^, which is often treated with chemotherapy, a limited number of studies have addressed the role of hematological indices in the prognosis of this advanced disease^22,24,26,29^. It is hypothesized that analyzing the association between the indices measured before chemotherapy and patient outcomes of those treated with a single first-line chemotherapy could yield a prognostic model that is more robust and applicable to these patients.

Evaluating immune cells in the tumor microenvironment (TME) can also be used to estimate the outcome of cancer patients. The presence of tumor-infiltrating lymphocytes (TILs) or tumor-associated macrophages (TAMs) in the TME impacts the prognosis of patients^32–39^. In patients with hepatocellular carcinoma undergoing liver transplantation, the blood LMR correlated with the CD3-positive to CD68-positive cell ratio (potentially TILs to TAMs ratio) in the resected specimens^40^. Moreover, NLR inversely correlated with the density of CD8-positive TILs in lung cancer^41^. These findings suggest that anti-tumor immunity in the TME could be noninvasively monitored through these hematological indices.

In this study, we primarily determined which of the indices (LMR, NLR, and PLR) was most associated with increased survival in patients with advanced STS treated with first-line DXR therapy, and secondarily explored the relationship between the prognostic index and the histopathological TME status.

## Results

### Patient characteristics

Between August 2009 and December 2018, 153 patients were treated with first-line DXR at the National Cancer Center Hospital. Since four patients with missing differential WBC counts were excluded, 149 patients were enrolled in the study. Among these patients, 145 experienced disease progression (PD) before the day of data cut-off.

The patients’ characteristics are summarized in Table 1. Eleven patients (7.4%) had Eastern Cooperative Oncology Group performance status (PS) of 2 or 3. The primary tumor was resected and recurred in 108 (72.5%) patients. Tumors were classified as abdominal or thoracic visceral in 74 patients (49.7%) according to the American Joint Committee on Cancer (AJCC) staging system 8th edition^12^. Leiomyosarcoma and liposarcoma affected 57 (38.3%) and 31 (20.8%) patients, respectively.

**Table 1:** Baseline patient characteristics.

Variables	Total n = 149 (%)
Age median (range)	53 (15–78)
Gender	
Female/Male	94 (63.1)/55 (36.9)
ECOG PS	
0	75 (50.3)
1	63 (42.3)
2	9 (6.1)
3	2 (1.3)
Charlson Comorbidity Index Category	
Low/Medium	119 (79.9) / 30 (20.1)
Primary tumor resection*	
Yes/No	108 (72.5) / 41 (27.5)
Radiotherapy	
Yes/No	22 (14.8) / 127 (85.2)
Perioperative chemotherapy	
Yes/No	7 (4.7) / 142 (95.3)
Disease status	
Locally advanced (Inoperable)	21 (14.1)
Metastatic	63 (42.3)
Post-operative recurrence	65 (43.6)
Time to recurrence (months)	
≥ 10/< 10	33 (53.2)^†^/29 (46.8)^†^
Tumor site	
Visceral	74 (49.7)
Retroperitoneum	49 (32.9)
Trunk	14 (9.4)
Extremity	2 (1.3)
Others	10 (6.7)
Histology	
Leiomyosarcoma	57 (38.3)
Dedifferentiated liposarcoma	23 (15.4)
Undifferentiated pleomorphic sarcoma	19 (12.8)
Others^‡^	50 (33.5)
Metastasis site	
Lung/Liver/No	69 (46.3)^§^/42 (28.2)^§^/59 (39.6)
Pleural effusion	
Yes/No	19 (12.8)/130 (87.2)
Ascites	
Yes/No	63 (42.3)/86 (57.7)
Albumin (g/L)	
≥ 3.8/ < 3.8	90 (60.4)/59 (39.6)
LDH (U/L)	
≥ 246/ < 246	43 (28.9)/106 (71.1)
CRP (mg/dL)	
< 0.31/ ≥ 0.31	61 (40.9)/88 (59.1)
LMR	
≥ 2.1 / < 2.1	125 (83.9)/24 (16.1)
NLR	
≥ 5.5 / < 5.5	33 (22.1)/116 (77.9)
PLR	
≥ 264 / < 264	56 (37.6)/93 (62.4)

CRP, c-reactive protein; ECOG PS, Eastern Cooperative Oncology Group performance status; LDH, lactate dehydrogenase; LMR, lymphocyte-to-monocyte ratio; NLR, neutrophil-to-lymphocyte ratio; OS, overall survival; PLR, platelet-to-lymphocyte ratio; PFS, progression-free survival.

*Tumor resection includes curative and debulking surgery.

^†^Among 65 patients with postoperative recurrence, the time from surgery to recurrence was not available in three patients.

^‡^The other histologies include the follows: malignant spindle cell sarcoma, 7; intimal sarcoma, 5; desmoplastic small round cell tumor, 4; undifferentiated spindle cell sarcoma, 4; endometrial stromal sarcoma, 3; synovial sarcoma 3; unclassifiable sarcoma, 3; chondrosarcoma, 2; epithelioid hemangioendothelioma, 2; malignant peripheral nerve sheath tumor, 2; pleomorphic spindle cell sarcoma, 2; solitary fibrous tumor, 2; well-differentiated liposarcoma, 2; breast stromal sarcoma, 1; epithelioid sarcoma, 1; Ewing sarcoma, 1; inflammatory myofibroblastic tumor, 1; malignant myoepithelioma, 1; round cell sarcoma, 1; SMARCA4-deficient sarcoma, 1; undifferentiated uterine sarcoma, 1; uterine adenosarcoma, 1.

^§^Twenty-one patients had metastasis in the lung and liver.

### Variation of the hematological indices

**Figure 1:**
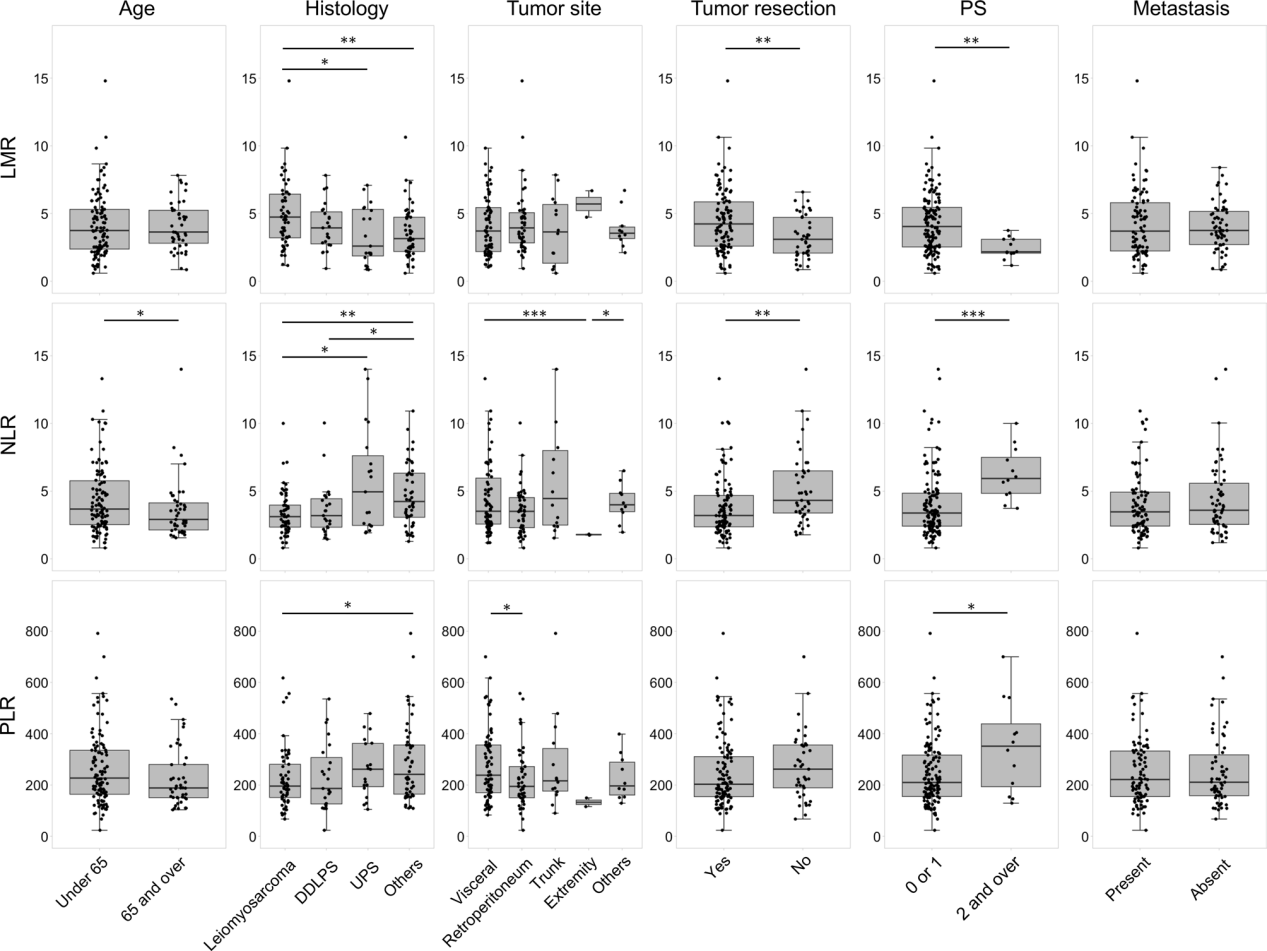
Variation in hematological indices among selected patient subgroups. Box plots showing indices according to age, histology, tumor site, presence of primary tumor resection, PS, and presence of metastasis. Each point on the scatter plot represents an individual patient within a specified subgroup. The overlaid box plot presents the median and interquartile range of the indices for all patients. DDLPS, dedifferentiated liposarcoma; LMR, lymphocyte-to-monocyte ratio; NLR, neutrophil-to-lymphocyte ratio; PLR, platelet-to-lymphocyte ratio; PS, performance status; UPS, undifferentiated polymorphic sarcoma. *p < 0.05; **p < 0.01; ***p < 0.001.

Interpatient variation in the hematological indices among the selected subgroups is depicted in Fig. 1. Patients who relapsed after primary tumor resection had a significantly higher baseline LMR (4.47 vs. 3.27, *p* = 0.004) and lower baseline NLR (4.00 vs. 5.15, *p* = 0.001) than those who did not receive surgery. Patients with PS of 0 or 1 had higher LMR (4.29 vs. 2.45, *p* = 0.002), lower NLR (4.15 vs. 6.27, *p* = 0.0003), and lower PLR (245.3 vs. 350.2, *p* = 0.044) than patients with higher PS. The pairwise correlations between each of the variables are shown in Supplementary Fig. 1. Notably, LMR negatively correlated with PS (ρ = − 0.43), NLR (− 0.71), PLR (− 0.61), lactose dehydrogenase (LDH) (− 0.28), and C-reactive protein (CRP) (− 0.65) values, and positively correlated with albumin (0.57) levels.

### Prognostic value of LMR for overall survival

The optimal cutoff values for the hematological indices were determined using receiver operating characteristic (ROC) analyses. Based on Youden’s index, the cutoff values for the LMR, NLR, and PLR in predicting OS were 2.1, 5.5, and 264.0, respectively. To estimate PFS, the cutoff values for the LMR, NLR, and PLR were set to 3.3, 4.3, and 239.0, respectively.

**Figure 2:**
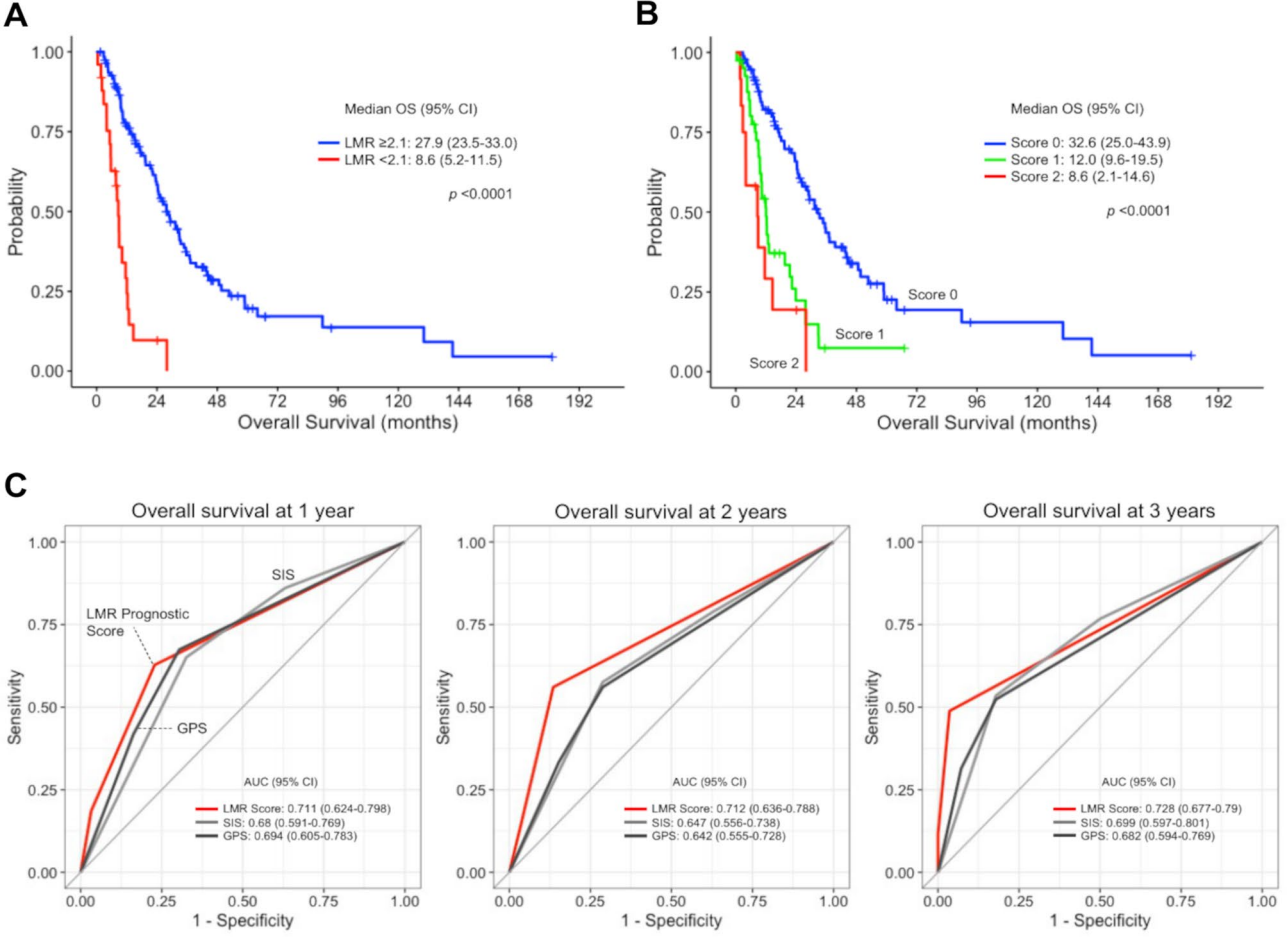
OS stratified by LMR (**A**) and the LMR prognostic score (**B**). (**C**) The prognostic potential of the LMR prognostic score was compared with that of SIS or GPS in ROC analyses for OS at 1 year (left), 2 years (middle), and 3 years (right). AUC, area under curve; CI, confidence interval; DXR, doxorubicin; GPS, Glasgow prognostic score; LMR, lymphocyte-to-monocyte ratio; OS, overall survival; SIS, Systemic Inflammatory Score.

In the Cox regression model, univariate analysis showed that low LMR (hazard ratio [HR] 6.17, *p* < 0.0001), high NLR (HR 2.68, *p* = 0.0001), and high PLR (HR 2.92, *p* < 0.0001) were associated with worse OS. Furthermore, lower OS was also associated with a PS ≥ 2 (HR 2.42, *p* = 0.019), absence of primary tumor resection (HR 2.21, *p* = 0.0008), decreased albumin level (HR 1.98, *p* = 0.001), and elevated LDH (HR 2.07, *p* = 0.002) and CRP (HR 2.39, *p* < 0.0001) levels (Table 2). Multivariate analysis indicated that low LMR (HR 3.93, *p* = 0.001) and absence of primary tumor resection (HR 1.71, *p* = 0.034) were independent risk factors for OS. In the Kaplan–Meier analysis, the median OS for the overall study population was 24.3 months (Supplementary Fig. 2A), and patients with a low LMR (8.6 vs. 27.9 months, *p* < 0.0001) (Fig. 2A), a high NLR (8.9 vs. 27.9 months, *p* < 0.0001), and a high PLR (10.1 vs. 32.3 months, *p* < 0.0001) had shorter OS than their respective opposing groups (Supplementary Fig. 2B).

**Table 2:** Univariate and multivariate Cox regression analysis for OS.

Variables	Univariate analysis		Multivariate analysis	
	HR (95% CI)	*p*-value	HR (95% CI)	*p*-value
Age				
< 65	1.0	0.297		
≥ 65	1.27 (0.80–1.95)			
Gender				
Female	1.0	0.692		
Male	0.92 (0.60–1.38)			
ECOG PS				
0 or 1	**1.0**	**0.019**	1.0	0.209
≥ 2	**2.42 (1.17–4.49)**		1.64 (0.74–3.32)	
CCI Category				
Low	1.0	0.974		
Medium	0.99 (0.58–1.60)			
Primary tumor resection*				
Yes	**1.0**	**0.0008**	**1.0**	**0.034**
No	**2.21 (1.41–3.39)**		**1.71 (1.04–2.75)**	
Radiotherapy				
Yes	1.0	0.190		
No	1.43 (0.85–2.59)			
Perioperative chemotherapy				
Yes	1.0	0.581		
No	1.36 (0.51–5.57)			
Disease status				
Locally advanced	1.0			
Metastatic	1.63 (0.88–3.32)	0.125		
Recurrence	0.76 (0.41–1.56)	0.434		
Time to recurrence (months)				
≥ 10	1.0	0.127		
< 10	1.65			
Tumor site				
Trunk	1.0			
Visceral	0.97 (0.50–2.11)	0.939		
Retroperitoneum	0.48 (0.23–1.09)	0.075		
Extremity	0.79 (0.12–3.09)	0.761		
Others	0.98 (0.33–2.73)	0.972		
Histology				
Leiomyosarcoma	1.0			
DDLPS	0.92 (0.47–1.69)	0.614		
UPS	1.84 (0.79–3.77)	0.181		
Others^†^	1.59 (0.97–2.49)	0.062		
Metastasis				
No	1.0			
Lung	0.95 (0.64–1.42)	0.812		
Liver	0.90 (0.58–1.38)	0.644		
Pleural effusion				
No	1.0	0.378		
Yes	1.33 (0.69–2.34)			
Ascites				
No	1.0	0.094		
Yes	1.41 (0.94–2.11)			
Albumin (g/L)				
≥ 3.8	**1.0**	**0.001**	1.0	0.973
< 3.8	**1.98 (1.32–2.95)**		1.01 (0.62–1.64)	
LDH (U/L)				
< 246	**1.0**	**0.002**	1.0	0.740
≥ 246	**2.07 (1.32–3.18)**		1.09 (0.65–1.79)	
CRP (mg/dL)		** < 0.0001**		0.261
< 0.31	**1.0**		1.0	
≥ 0.31	**2.39 (1.58–3.68)**		1.37 (0.79–2.38)	
LMR				
≥ 2.1	**1.0**	** < 0.0001**	**1.0**	**0.001**
< 2.1	**6.17 (3.46–10.66)**		**3.93 (1.73–9.17)**	
NLR				
< 5.5	**1.0**	**0.0001**	1.0	0.644
≥ 5.5	**2.68 (1.66–4.18)**		0.85 (0.41–1.67)	
PLR				
< 264	**1.0**	** < 0.0001**	1.0	0.063
≥ 264	**2.92 (1.93–4.39)**		1.71 (0.97–3.01)	

CCI, Charlson Comorbidity Index; CRP, c-reactive protein; DDLPS, dedifferentiated liposarcoma; ECOG PS, Eastern Cooperative Oncology Group performance status; LDH, lactate dehydrogenase; LMR, lymphocyte-to-monocyte ratio; NLR, neutrophil-to-lymphocyte ratio; OS, overall survival; PLR, platelet-to-lymphocyte ratio; UPS, undifferentiated pleomorphic sarcoma.

*Tumor resection includes curative and debulking surgery.

^†^The other histologies include the follows: malignant spindle cell sarcoma, 7; intimal sarcoma, 5; desmoplastic small round cell tumor, 4; undifferentiated spindle cell sarcoma, 4; endometrial stromal sarcoma, 3; synovial sarcoma 3; unclassifiable sarcoma, 3; chondrosarcoma, 2; epithelioid hemangioendothelioma, 2; malignant peripheral nerve sheath tumor, 2; pleomorphic spindle cell sarcoma, 2; solitary fibrous tumor, 2; well-differentiated liposarcoma, 2; breast stromal sarcoma, 1; epithelioid sarcoma, 1; Ewing sarcoma, 1; inflammatory myofibroblastic tumor, 1; malignant myoepithelioma, 1; round cell sarcoma, 1; SMARCA4-deficient sarcoma, 1; undifferentiated uterine sarcoma, 1; uterine adenosarcoma, 1.

Significant values are in bold.

### Development of LMR prognostic score

Based on the results of the multivariate analysis, we developed a prognostic scoring model utilizing LMR and a history of primary tumor resection (Table 3). Patients with an LMR of less than 2.1 or those who did not undergo primary tumor resection had a partial score of 1, which yielded a total score ranging from 0 to 2. As shown in Table 4**,** and in common with SIS and GPS**,** the LMR-based model was associated with worse OS (score of 1, HR 2.26, *p* = 0.004; score of 2, HR 8.09, *p* < 0.0001). Kaplan–Meier analysis showed that compared with patients with a prognostic score of 0, those with scores of 1 and 2 had shorter OS (median 32.6, 12.0, and 8.6 months, *p* < 0.0001) (Fig. 2B). ROC analysis showed that the area under curves (AUCs) for LMR prognostic score were 0.711 (95% CI, 0.624 to 0.798), 0.712 (0.636 to 0.788), and 0.728 (0.677–0.79) for 1-year, 2-year, and 3-year survival, respectively (Fig. 2C). To internally validate the LMR-based scoring model, we performed a resampling analysis using the bootstrap method (that is, the model was resampled 1000 times) and found minimal overfitting (mean values were overoptimistic by 0.0005 for 1-year survival, 0.0002 for 2-year survival, and 0.0004 for 3-year survival). Although there was no significant statistical difference, the LMR-based scoring model showed larger AUCs than those of Systemic Inflammation Score (SIS)^42^ or Glasgow Prognostic Score (GPS)^43^ across 1 year, 2-year, and 3-year survival (Supplementary Table 1), which indicates that discrimination accuracy of the LMR prognostic score is sufficient.

**Table 3:** Calculation of LMR prognostic score.

Variables	Patients	HR (95% CI)	Partial score
LMR			
≥ 2.1	124	1.0	0
< 2.1	25	3.93 (1.73–9.17)	1
Primary tumor resection			
Yes	108	1.0	0
No	41	1.71 (1.04–2.75)	1

Score is calculated as follows: LMR prognostic score = LMR score + primary tumor resection score. CI, confidence interval; LMR, lymphocyte-monocyte ratio.

**Table 4:** Cox regression analysis for OS using LMR prognostic score, SIS, and GPS.

Prognostic model	Patient	Univariate analysis	
		HR (95% CI)	*p*-value
LMR prognostic score			
0	95	**1.0**	
1	42	**2.26 (1.32–3.73)**	**0.004**
2	12	**8.09 (3.77–16.3)**	** < 0.0001**
SIS			
0	45	**1.0**	
1	41	1.13 (0.66–1.93)	0.661
2	63	**2.40 (1.50–3.92)**	**0.0002**
GPS			
0	85	**1.0**	
1	26	**1.85 (1.06–3.09)**	**0.031**
2	38	**2.62 (1.62–4.16)**	**0.0001**

CI, confidence interval; GPS, Glasgow prognostic score; LMR, lymphocyte-monocyte ratio; OS, overall survival; SIS, Systemic Inflammation Score.

Significant values are in bold.

### Predictive value of LMR for the efficacy of DXR therapy

The median PFS of patients treated with DXR therapy was 4.3 months (Supplementary Fig. 3A). Those with low LMR (3.1 vs. 5.3 months, *p* = 0.005), high NLR (3.3 vs. 4.9 months, *p* = 0.011), and high PLR (3.2 vs. 5.5 months, *p* = 0.004) possessed shorter PFS (Supplementary Fig. 3B). However, in the multivariate Cox regression analysis no indices were significantly associated with PFS (low LMR, HR 1.09, *p* = 0.721; high NLR, HR 1.06, *p* = 0.792; high PLR, HR 1.01, *p* = 0.972) (Supplementary Table 2) and the efficacy of DXR therapy (LMR, *p* = 0.113; NLR, *p* = 0.521; PLR, *p* = 0.507) (Supplementary Fig. 3C). Together, these data suggest a limited predictive value of the indices for the efficacy of DXR therapy.

### Association between LMR and TME

**Figure 3:**
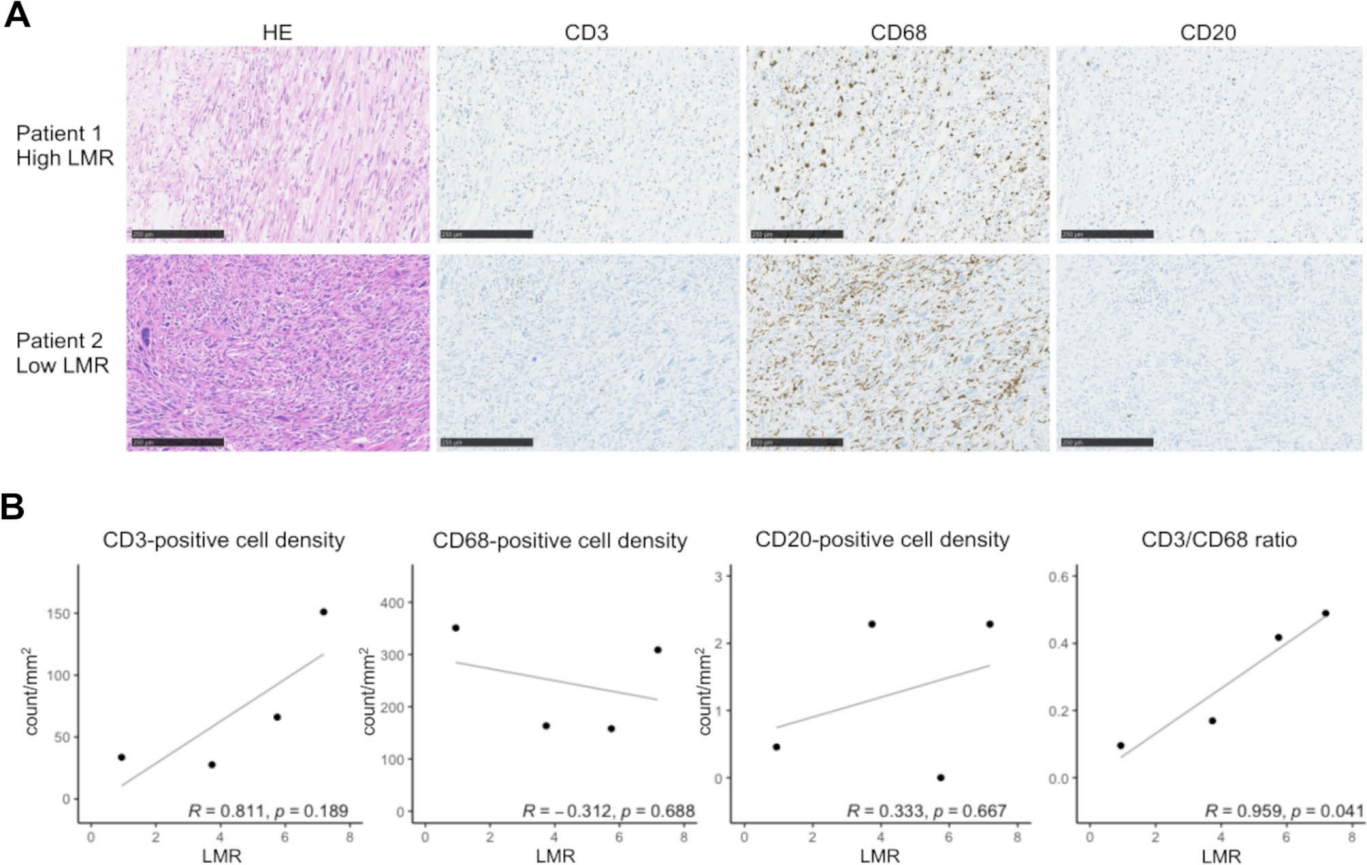
(**A**) Immunohistochemical analysis of the selected surgical samples. The upper images show a leiomyosarcoma with a high LMR of 7.19. The HE-stained (left), CD3-positive (middle left), CD68-positive (middle right), and CD20-positive (right) cells are shown. The lower images show a dedifferentiated liposarcoma with a low LMR of 0.94. Scale bars: 250 μm. (**B**) Correlation between LMR and the densities of positive cells. Pearson correlation coefficients are shown. HE, hematoxylin and eosin; LMR, lymphocyte-to-monocyte ratio.

To explore whether the LMR correlated with TILs status in the TME, we performed immunohistochemical (IHC) analysis for CD3, CD68, and CD20 using surgical specimens (Fig. 3A). As a pilot analysis, we selected four representative samples of leiomyosarcoma and liposarcoma from 26 cases with available surgical samples. The CD3-positive cells were more abundant in the high-LMR tumor (upper images) than in the low-LMR tumor (lower images), whereas the CD68-positive cells were less abundant in the high-LMR tumor (upper images) than in the low-LMR tumor (lower images). The correlations between the LMR and the densities of tumor-infiltrating immune cells are shown in Fig. 3B. LMR significantly correlated with the tumoral CD3/CD68 ratio (R = 0.959, *p* = 0.041).

## Discussion

In this study, we evaluated the roles of three hematological indices (LMR, NLR, and PLR) in the prognosis of advanced STS and identified the predictive value of the LMR measured before DXR therapy. Overall, low LMR was independently associated with shorter OS, and the LMR-based scoring model was able to discriminate high-risk patients. Furthermore, the LMR correlated with the tumoral CD3/CD68 ratio in the surgical samples, suggesting that the LMR partly reflects anti-tumor immunity governing the TME.

Staging systems, such as the AJCC TNM system, are widely used in the prognosis of patients with STS^12^. However, the separation of patients into accurate prognostic cohorts in this way is hindered by the histological diversity of STS^44^, prompting the identification of new prognostic factors outside of the nature of the tumor itself. Studies have reported that age, chemotherapeutic regimen, PS, and levels of albumin, CRP, and hemoglobin are prognostic factors for survival in patients with advanced STS^45–50^. Additionally, GPS, a cumulative score based on CRP and albumin levels^43^, was also prognostic for OS in patients with advanced STS^51^. While hematological indices were prognostic for STS^17–31^, most of these studies only considered resectable STS and analyzed preoperative parameters, with a focus on the NLR^17–21,23,25,27^. Finally, some studies on these prognostic factors have excluded these indices in survival analysis altogether^45–50^. Our study revealed the prognostic value of the LMR for patients with advanced STS treated with DXR, which is in line with previous findings that the LMR or peripheral monocyte ratio is prognostic for OS in patients with metastatic STS^21,22^.

In this study, the LMR level was associated with the tumoral CD3/CD68 ratio. The mechanism behind this relationship may be partly explained in terms of the TME. Accumulating evidence shows that the TME dynamically regulates tumor progression and influences therapeutic outcomes^52^. Some TILs function as cytotoxic T cells that suppress tumor growth, and a high density of TILs has been linked to improved clinical outcomes in solid tumors^53–55^. TAMs are partly derived from circulating monocytes and recruited to the tumor site by tumor-derived chemotactic factors. TAMs can inhibit cytotoxic T cell responses through immune checkpoint engagement, production of cytokines, metabolic activities, and modulation of the TME^56^. Patients with high infiltration of TAMs had worse OS^57,58^. The peripheral monocyte count can be representative of the TAMs because a high peripheral monocyte count is a major risk factor in patients with solid tumors^59^ and is associated with a high density of TAMs^60^. Given the opposing functions of TILs and TAMs, it is plausible that the LMR can represent the dynamic equilibrium between immune cells in the TME. The correlation between LMR and CD3/CD68 ratio has also been reported in patients with hepatocellular carcinoma^40^. Collectively, low LMR was associated with worse survival, possibly due to an insufficient anti-tumor immune response in the TME.

The use of DXR therapy in our cohort may be another possible reason for the prognostic role of the LMR. DXR drives immunogenic cell stress by activating an adaptive immune response and eliciting immunological memory in immunocompetent hosts, resulting in long-lasting protective antitumor immunity^61–64^. This immune response can only be properly executed in a permissive TME that contains abundant cytotoxic TILs and/or scarce immunosuppressive TAMs^61^. In this context, tumors with higher CD3/CD68 ratios might favor the occurrence of immunogenic cell stress and be more sensitized to cytotoxic T cells, which could be the reason for longer survival after DXR therapy in high-LMR patients.

Contrary to the prognostic value of the LMR, its predictive value for the efficacy of DXR therapy remains unclear because the LMR was not associated with PFS or treatment response in our study. The mode of drug-induced immune response can partly explain the reason for this discrepancy. Trabectedin, an approved anticancer agent, not only triggers cell-cycle arrest and apoptosis in tumor cells but also depletes TAMs in the TME^65,66^. Its immunomodulatory property has been thought to elicit a delayed response with prolonged stabilization of disease^67^. Indeed, in a phase 3 trial of advanced STS, most patients who benefited from trabectedin experienced durable stable disease rather than tumor shrinkage^9^. PFS and treatment response indicate an early therapeutic efficacy compared to OS and thus cannot encompass the long-term aspect of DXR-induced immune response, which might lead to better OS in high-LMR patients. Additionally, the inhibition of the vascular endothelial growth factor (VEGF) pathway normalizes the tumor blood vessels and reprograms the immunosuppressive TME into an immunostimulatory milieu in solid tumors^68^. Pazopanib, a VEGF receptor inhibitor, induces immune activation by influencing the differentiation and maturation of dendritic cells^69^. Future studies should highlight the prognostic and predictive role of LMR, characterizing the TME with more phenotypic markers of TILs and TAMs in patients treated with immunomodulatory anticancer agents.

Primary tumor resection was independently associated with a longer OS and higher LMR. Moreover, patients with resected STS had higher LMRs and NLRs before DXR therapy than those with unresected STS, suggesting that primary tumor resection impacted immunological control of the disease^16^. Tumor resection is thought to induce immunosuppression after surgery^70^. However, recent studies have shown that successful tumor resection largely reverts systemic immune dysfunction, including the cytotoxicity of T cells, and restored immunocompetence, even in patients with metastatic disease^71,72^. Theoretically, primary tumor resection could rescue the systemic immunity and facilitate the adaptive immune response, potentially augmented by DXR therapy. This could contribute to better survival after DXR therapy in patients receiving surgery than those who did not. Another possible reason for the favorable surgical result is the paucity of effective systemic therapy for advanced STS. For patients with metastatic renal cell carcinoma, cytoreductive nephrectomy has long been the sole standard of care. However, a phase 3 trial has shown that sunitinib alone was not inferior to cytoreductive nephrectomy, followed by sunitinib, in patients who were classified as having intermediate- or poor-risk disease^73^, and only patients with good PS and local symptoms are currently recommended to undergo upfront surgery^74^. This suggests that the efficacy of promising systemic therapies outweighs the survival advantage of surgery. However, no promising therapy has been introduced for patients with advanced STS. Our study emphasized the significance of primary tumor resection for patients with STS.

The LMR prognostic score, based on the LMR level and tumor resection history, reliably identified high-risk patients. The accurate prediction of survival can help future care and provide opportunities for patients and their families to focus on what is relevant to them when time is limited^75^. Our model can help avoid harm and inappropriate therapies in vulnerable patients^76^ and enhance patients’ autonomy^77^. In addition to being a prognostic estimate, the LMR score can offer an insight into the TME status. The inferred TME status has been reported to be associated with outcomes in multiple types of sarcomas^78^ and predictive of response to immunotherapy^79^. Thus, the LMR scoring model can facilitate decision making regarding optimal treatment.

This study was limited by its retrospective nature as well as the enrollment of patients from only a single institution. Since we included patients with unresectable and recurrent STS treated with DXR therapy, we had a limited number of surgical samples available and could not fully capture the pathological data on the specimens. The opportunity to sample additional tumor tissues before chemotherapy was limited by a local health insurance scheme in clinical practice. Additionally, data on the tumor grade were lacking. According to the definition of the La Fédération Nationale des Centres de Lutte Contre le Cancer grading system^80,81^, we scored only 10 of the 26 resected samples available and thus excluded the grade in the survival analysis. We obtained the results of IHC analysis from only a few resected samples because we excluded biopsied samples from staining and secondarily evaluated the relationship between the LMR and TME status. The IHC results from the resected tumor samples reflected the TME status at the time of primary tumor resection, which might have been altered before DXR therapy. We enrolled patients treated with DXR therapy, excluding other types of chemotherapy, because DXR has been the most common agent for advanced STS among the few drugs covered by the local healthcare system. Differences in characteristics between patients treated with DXR and those treated with other agents (paclitaxel and pazopanib) during the study period are not shown because some data were unavailable. Our patient selection according to the treatment regimen introduced results that should be generalized with caution to all patients with advanced STS. Finally, although the benefit of histology-tailored chemotherapy for STS has been debatable^82,83^, developing subtype-specific or biomarker-driven strategies is essential. The diverse tumor histologies affected the variation of the indices; thus, the results should be interpreted cautiously.

In conclusion, the LMR was an independent prognostic factor for OS in patients with advanced STS treated with first-line DXR therapy, and the LMR prognostic scoring model reliably predicted OS. Additionally, measuring peripheral LMR can provide clues to the anti-tumor immunity status in the TME. Further investigation is needed to examine the underlying mechanisms of the relationship between the LMR and TME.

## Patients and methods

### Patients

Retrospectively, we reviewed patients with unresectable or recurrent STS who received first-line DXR therapy at the National Cancer Center Central Hospital between August 2009 and December 2018. The eligibility criteria for this study were histologically confirmed STS and availability of laboratory data that were measured before DXR therapy and included differential WBC counts. This study was approved by the ethics committee of the National Cancer Center Hospital (approval numbers: 2012-335, 2016-086). Informed consent was obtained from all participants and/or their legal guardians. All methods were performed in accordance with the relevant guidelines and regulations.

### Clinical characteristics and hematological indices

Clinical characteristics and laboratory data before DXR therapy were taken from the medical records. The parameters collected included patients’ age, sex, PS before DXR therapy, and comorbidity; history of primary tumor resection, radiotherapy, and perioperative (adjuvant and neoadjuvant) chemotherapy prior to first-line DXR therapy, disease status, and time to recurrence; primary tumor site; histological type of STS; and presence of metastasis, pleural effusion, and ascites. Laboratory data included absolute counts of lymphocytes, neutrophils, monocytes, and platelets, as well as albumin, LDH, and CRP levels.

LMR, NLR, and PLR were defined as the ratio of absolute lymphocyte count divided by absolute monocyte count, absolute neutrophil count divided by absolute lymphocyte count, and absolute platelet count divided by absolute lymphocyte count, respectively. Optimal cutoffs for the indices were determined using Youden’s index in ROC analyses. Cutoff values for albumin, LDH, and CRP were determined as the upper and lower limits of the normal ranges for the variables in our institution.

The Charlson Comorbidity Index^84^, SIS^42^, and GPS^43^ were calculated as previously described, and patients were categorized into subgroups according to their indices.

### Patient outcomes

The tumor was evaluated using computed tomography (CT) before DXR therapy and again after two or three cycles of DXR therapy. After the first evaluation, CT scans were performed when clinically necessary. The efficacy of DXR therapy was determined according to the Response Evaluation Criteria in Solid Tumors, version 1.1^85^.

OS was defined as the period from the date of pathological diagnosis of STS in patients with unresectable tumors or from the date of recurrence in patients with resected tumors to the date of death from any cause or the last follow-up. PFS was defined as the period from the date of initiation of DXR therapy to the date of documentation of PD, death from any cause, or the last follow-up. Data cut off occurred on November 30, 2021.

### Immunohistochemistry

Tissue specimens from patients with STS were available from pathology files at the National Cancer Center. All the tissues were formalin-fixed and embedded in paraffin (FFPE). The histology of each case was evaluated by a board-certified sarcoma pathologist (A.Y.). If patients experienced postoperative recurrence during follow-up in external hospitals and were referred to us, the diagnosis of STS subtypes was confirmed using the external FFPE samples. Diagnostic rebiopsy or tumor resection was performed only if clinically required. IHC analysis of the tumor was performed in resected samples because we excluded biopsied samples from being immunohistochemically stained and secondarily evaluated the relationship between the LMR and TME status. The IHC staining was performed according to the manufacturer’s instructions using an autostainer (Dako Autostainer Link 48 and Omnis staining platform; Dako, Glostrup, Denmark) and the following monoclonal antibodies: CD3 (PS1, Leica Biosystems, Nussloch, Germany), CD68 (PGM1, Dako), and CD20 (L26, Thermo Fisher Scientific, MA, US).

### Evaluation of tumor-infiltrating immune cells

The stained slides were examined using a high-resolution digital slide scanner (NanoZoomer 2.0-HT whole-slide imager; Hamamatsu Photonics, Hamamatsu, Japan). Immune cell infiltration was evaluated by two observers (S.W. and A.Y.). Seven independent 0.0625-mm^2^ areas containing immune cells in the central tumor were selected, and the number of positively-stained immune cells was averaged for each patient. The density of tumor-infiltrating immune cells was calculated as the average number of stained cells divided by the examined area (per 1 square millimeter). The CD3/CD68 ratio was calculated as the density of CD3-positive cells divided by the density of CD68-positive cells.

### Statistical analysis

Variation in hematological indices among patient subgroups was compared using the Wilcoxon rank-sum test. The correlation between each laboratory data and PS was estimated using a Spearman’s rank correlation coefficient. The correlation between the indices and the densities of tumor-infiltrating immune cells was analyzed using Pearson’s correlation coefficients. Association of the indices with the efficacy of DXR therapy was tested using the chi-squared test.

OS and PFS were examined using the Kaplan–Meier method and differences in survival between patient subgroups were compared using the log-rank test. HRs and 95% confidence intervals were estimated using the Cox regression analysis. All significant variables identified by univariate analysis were further evaluated by multivariate analysis. Based on the results of multivariate analysis, a prognostic model for survival was constructed and internally validated by resampling using the bootstrap method^86^. The prognostic performance of the model was evaluated by calculating the area AUC in the ROC analysis, and the difference in AUCs between the models was examined using the DeLong test.

Statistical analyses were performed using JMP version 12.0.1 (SAS Institute Inc., NC, US) and R ver. 4.0.2 (R Foundation for Statistical Computing, Vienna, Austria). All statistical tests were two-sided, and a *p*-value less than 0.05 was regarded as statistically significant.

## Supplementary Information

Supplementary Information 1.

## Data Availability

The datasets generated during and/or analyzed during the current study are not publicly available due to sensitivity of human data but are available from the corresponding author on reasonable request.
