# Supplementary material for: Lymphocyte-to-monocyte ratio as a prognostic and potential tumor microenvironment indicator in advanced soft tissue sarcoma treated with first-line doxorubicin therapy
Source: Sci Rep. 2023 Jul 3;13:10734. doi: 10.1038/s41598-023-37616-w (PMC10318000; doi:10.1038/s41598-023-37616-w)
Supplement: Supplementary file 1 — Supplementary Information 1. [file 41598_2023_37616_MOESM1_ESM.pdf]

Supplementary Materials for

Lymphocyte-to-monocyte ratio as a prognostic and potential tumor microenvironment indicator in advanced soft tissue sarcoma treated with first-line doxorubicin therapy

Sho Watanabe, Tatsunori Shimoi*, Tadaaki Nishikawa, Asuka Kawachi, Hitomi Sumiyoshi Okuma, Momoko Tokura, Shu Yazaki, Chiharu Mizoguchi, Motoko Arakaki, Ayumi Saito, Shosuke Kita, Kasumi Yamamoto, Yuki Kojima, Kazuki Sudo, Emi Noguchi, Akihiko Yoshida, Akira Kawai, Yasuhiro Fujiwara, Kan Yonemori

* Corresponding author.
Correspondence to:
Tatsunori Shimoi, M.D., Ph.D.,
Department of Medical Oncology, National Cancer Center Hospital, 1-1, Tsukiji 5, Chuo-ku, Tokyo 104-0045, Japan.
Phone: +81-3-3542-2511, FAX: +81-3-3542-3815.
E-mail: tshimoi@ncc.go.jp

This PDF file includes:

Table S1 and S2
Figure S1, S2, and S3

Table S1. Prognostic potential of the LMR prognostic score, SIS, and GPS.

Outcome	Model	AUC (95% CI)	*p*-value (versus LMR score)
1-year survival	LMR prognostic score	0.711 (0.624-0.798)	-
	SIS	0.68 (0.591-0.769)	0.531
	GPS	0.694 (0.605-0.783)	0.719
2-year survival	LMR prognostic score	0.712 (0.636-0.788)	-
	SIS	0.647 (0.556-0.738)	0.176
	GPS	0.642 (0.555-0.728)	0.113
3-year survival	LMR prognostic score	0.728 (0.677-0.79)	-
	SIS	0.699 (0.597-0.801)	0.563
	GPS	0.682 (0.594-0.769)	0.325

AUC, area under curve; CI, confidence interval; GPS, Glasgow Prognostic Score; LMR, lymphocyte-to-monocyte ratio; SIS, Systemic Inflammatory Score.

Table S2. Univariate and multivariate Cox regression analysis for PFS

**Variables**	**Univariate analysis**		**Multivariate analysis**	
	HR (95% CI)	*p*-value	HR (95% CI)	*p*-value
Age				
<65	1.0	0.442		
≥65	1.16 (0.79-1.66)			
Gender				
Female	1.0	0.567		
Male	0.90 (0.63-1.27)			
ECOG PS				
0 or 1	1.0	0.112		
≥2	1.67 (0.88-2.92)			
CCI Category				
Low	1.0	0.196		
Medium	0.76 (0.49-1.15)			
Primary tumor resection*				
Yes	1.0	0.134		
No	1.33 (0.91-1.90)			
Radiotherapy				
Yes	1.0	0.729		
No	0.92 (0.60-1.49)			
Perioperative chemotherapy				
Yes	1.0	0.305		
No	0.65 (0.32-1.55)			
Disease status				
Locally advanced	1.0			
Metastatic	0.94 (0.58-1.59)	0.820		
Recurrence	0.69 (0.43-1.16)	0.157		
Time to recurrence (months)				
≥10	1.0	0.127		
<10	1.65			
Tumor site				
Trunk	1.0			
Visceral	0.71 (0.41-1.32)	0.265		
Retroperitoneum	0.53 (0.30-1.01)	0.054		
Extremity	2.51 (0.39-9.14)	0.280		
Others	0.43 (0.18-1.00)	0.051		
Histology				
Leiomyosarcoma	1.0		1.0	
DDLPS	0.80 (0.46-1.32)	0.388	0.89 (0.50-1.53)	0.680
UPS	1.76 (0.88-3.25)	0.108	1.61 (0.86-2.89)	0.135
Others†	**1.72 (1.09-2.78)**	**0.010**	1.52 (0.98-2.35)	0.060
Metastasis				
No	1.0			
Lung	1.03 (0.73-1.43)	0.879		
Liver	0.95 (0.65-1.37)	0.804		

Table S2. (continued)

Variables	Univariate analysis		Multivariate analysis	
	HR (95% CI)	*p*-value	HR (95% CI)	*p*-value
Pleural effusion				
No	**1.0**	**0.001**		
Yes	**2.54 (1.49-4.11)**			
Ascites				
No	1.0	0.076		
Yes	1.35 (0.97-1.89)			
Albumin (g/L)				
≥3.8	**1.0**	**0.0008**	1.0	0.238
<3.8	**1.82 (1.28-2.55)**		1.29 (0.85-1.97)	
LDH (U/L)				
<246	**1.0**	**0.029**	1.0	0.408
≥246	**1.52 (1.04-2.18)**		1.22 (0.77-1.93)	
CRP (mg/dL)				
<0.31	**1.0**	**<0.0001**	1.0	0.183
≥0.31	**2.39 (1.58-3.68)**		1.34 (0.87-2.04)	
LMR				
≥3.3	**1.0**	**0.006**	1.0	0.821
<3.3	**1.63 (1.15-2.28)**		1.06 (0.64-1.76)	
NLR				
<4.3	**1.0**	**0.014**	1.0	0.667
≥4.3	**1.56 (1.10-2.19)**		1.11 (0.70-1.74)	
PLR				
<239	**1.0**	**0.006**	1.0	0.867
≥239	**1.63 (1.15-2.28)**		1.04 (0.65-1.67)	

CCI, Charlson Comorbidity Index; CRP, c-reactive protein; DDLPS, dedifferentiated liposarcoma; ECOG PS, Eastern Cooperative Oncology Group performance status; LDH, lactate dehydrogenase; LMR, lymphocyte-to-monocyte ratio; NLR, neutrophil-to-lymphocyte ratio; OS, overall survival; PLR, platelet-to-lymphocyte ratio; UPS, undifferentiated pleomorphic sarcoma.

* Tumor resection includes curative and debulking surgery.

† The other histologies include the follows: malignant spindle cell sarcoma, 7; intimal sarcoma, 5; desmoplastic small round cell tumor, 4; undifferentiated spindle cell sarcoma, 4; endometrial stromal sarcoma, 3; synovial sarcoma 3; unclassifiable sarcoma, 3; chondrosarcoma, 2; epithelioid hemangioendothelioma, 2; malignant peripheral nerve sheath tumor, 2; pleomorphic spindle cell sarcoma, 2; solitary fibrous tumor, 2; well-differentiated liposarcoma, 2; breast stromal sarcoma, 1; epithelioid sarcoma, 1; Ewing sarcoma, 1; inflammatory myofibroblastic tumor, 1; malignant myoepithelioma, 1; round cell sarcoma, 1; SMARCA4-deficient sarcoma, 1; undifferentiated uterine sarcoma, 1; uterine adenosarcoma, 1.

Figure S1

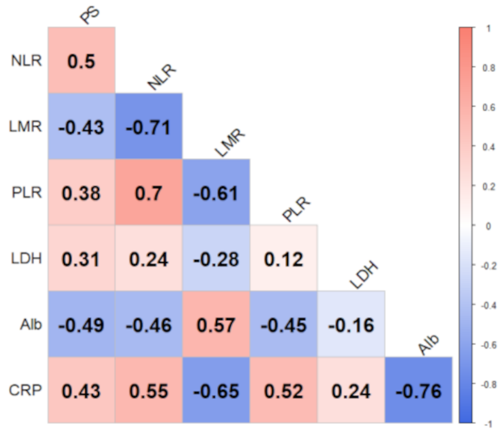

Pairwise correlations among the hematological indices, PS, and laboratory data obtained before doxorubicin therapy. Spearman rank correlation coefficients are noted.

Alb, albumin; CRP, c-reactive protein; LDH, lactose dehydrogenase; LMR, lymphocyte-to-monocyte ratio; NLR, neutrophil-to-lymphocyte ratio; PLR, platelet-to-lymphocyte ratio; PS, performance status.

Figure S2

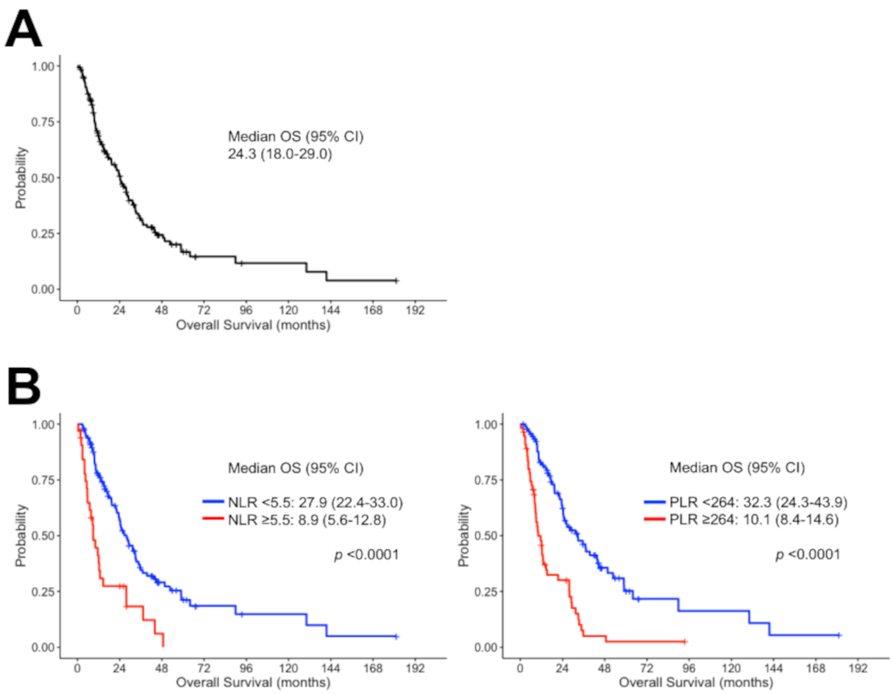

(A) OS of whole study population (n = 149). (B) OS stratified by NLR (left) and PLR (right).
CI, confidence interval; OS, overall survival; NLR, neutrophil-to-lymphocyte ratio; PLR, platelet-to-lymphocyte ratio.

Figure S3

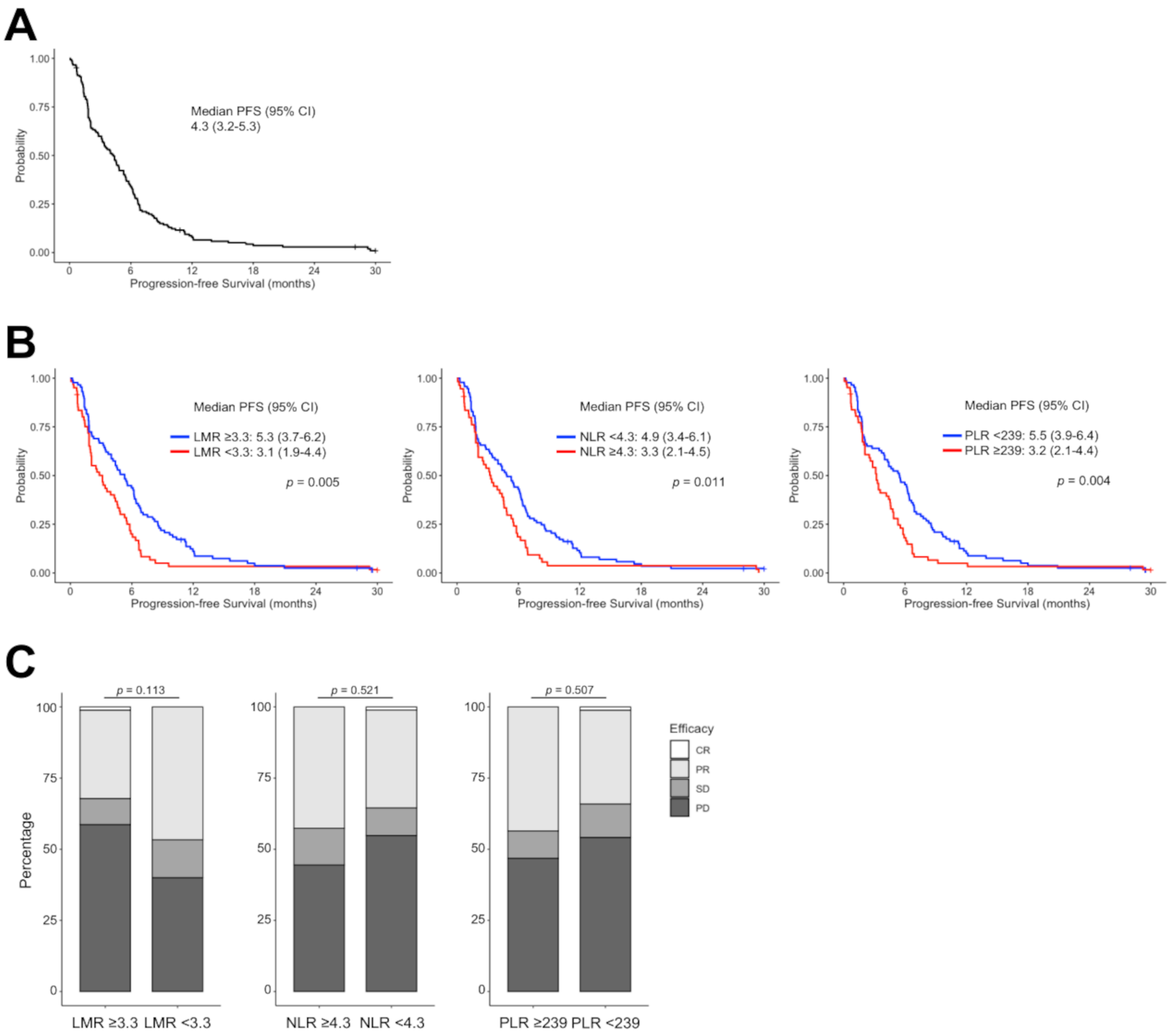

(A) PFS of whole study population (n = 149). (B) PFS stratified by LMR (left), NLR (middle), and PLR (right). (C) The efficacy of DXR therapy according to the index values.
CI, confidence interval; DXR, doxorubicin; LMR, lymphocyte-to-monocyte ratio; NLR, neutrophil-to-lymphocyte ratio; PLR, platelet-to-lymphocyte ratio; PFS, progression-free survival.
